# Mechanism of high affinity inhibition of the human urate transporter URAT1

**DOI:** 10.1038/srep34995

**Published:** 2016-10-07

**Authors:** Philip K. Tan, Traci M. Ostertag, Jeffrey N. Miner

**Affiliations:** 1 Department of Biology, Ardea Biosciences (A member of the AstraZeneca Group), 9390 Towne Centre Drive, San Diego, CA, 92121 USA

## Abstract

Gout is caused by elevated serum urate levels, which can be treated using inhibitors of the uric acid transporter, URAT1. We exploited affinity differences between the human and rat transporters to map inhibitor binding sites in URAT1. Human-rat transporter chimeras revealed that human URAT1 serine-35, phenylalanine-365 and isoleucine-481 are necessary and sufficient to provide up to a 100-fold increase in affinity for inhibitors. Moreover, serine-35 and phenylalanine-365 are important for high-affinity interaction with the substrate urate. A novel URAT1 binding assay provides support for direct interaction with these amino acids; thus, current clinically important URAT1 inhibitors likely bind the same site in URAT1. A structural model suggests that these three URAT1 residues are in close proximity potentially projecting within the channel. Our results indicate that amino acids from several transmembrane segments functionally cooperate to form a high-affinity URAT1 inhibitor binding site that, when occupied, prevents substrate interactions.

Gout is a metabolic disease caused by chronically elevated serum uric acid (sUA) levels (hyperuricemia) and deposition of urate in the joints, which leads to painful inflammatory arthritis^1^,^2^. Urate levels in the body are maintained by a balance between production and elimination. Hominoids and certain monkeys maintain relatively high sUA levels due to the presence of multiple inactivating mutations in the enzyme uricase^3^,^4^,^5^, which converts urate to allantoin in other animals. It is theorized that elevated sUA levels were selected during hominoid evolution^6^. Elimination of urate occurs primarily through the kidneys via a complex process of glomerular filtration, reabsorption and secretion^7^,^8^. Normally, approximately 90% of the glomerular-filtered urate is reabsorbed back into the bloodstream and approximately 10% is renally excreted. Most gout patients, however, exhibit enhanced reabsorption and reduced excretion of urate, leading to hyperuricemia. Other gout patients have elevated sUA due to enhanced production of urate. Gout therapies that lower sUA include those that inhibit the enzyme xanthine oxidase to block urate production (xanthine oxidase inhibitors or XOIs), as well as those that inhibit URAT1 to block renal urate reabsorption (URAT1 inhibitors or uricosurics) or enzymatically degrade uric acid (recombinant uricase)^9^,^10^.

Genome-wide association studies indicate that a large number of uric acid transporters are involved in urate homeostasis, including the solute carrier (SLC) transporters URAT1 (*SLC22A12*), organic anion transporter (OAT4), (*SLC22A11*), GLUT9 (*SLC2A9*), NPT1 (*SLC17A1*), NPT4 (*SLC17A3*), OAT2 (*SLC22A7*) and the ATP-binding cassette transporter ABCG2/BCRP (*ABCG2*)^11^,^12^,^13^,^14^,^15^. In the kidneys, glomerular-filtered urate is efficiently reabsorbed back into the bloodstream through the concerted activity of URAT1 and GLUT9a, located on the apical and basolateral sides, respectively, of proximal tubule epithelial cells^16^. Individuals carrying inactivating mutations of these genes have idiopathic renal hypouricemia and elevated fractional excretion of uric acid (FE_UA_), demonstrating the importance of these transporters in the renal reabsorption of uric acid^17^,^18^,^19^,^20^. NPT1, NPT4 and ABCG2, on the other hand, are involved in uric acid secretion based on biochemical and physiological studies, and genetic polymorphisms that reduce the activity of these transporters are associated with elevated sUA levels and gout^21^,^22^,^23^. The urate-lowering therapies probenecid, benzbromarone, sulfinpyrazone and lesinurad elevate FE_UA_ and lower sUA levels by inhibiting URAT1 in renal proximal tubules^18^,^24^. Currently, the use of the first three inhibitors for the treatment of gout varies between different countries^9^, while lesinurad (ZURAMPIC^®^) is a new URAT1 inhibitor approved in both Europe and the United States for the treatment of hyperuricemia associated with gout in combination with an XOI^25^.

The molecular mechanism of action of URAT1 inhibitors is unknown. Some compounds inhibit URAT1-mediated urate transport in a competitive manner^25^,^26^, suggesting inhibition through interaction with an undefined substrate recognition site. Functionally, URAT1 is an electroneutral organic anion exchanger, with a primary role in the inward transport of urate in exchange for the outward movement of monovalent organic anions such as lactate and nicotinate^18^. Structurally, URAT1 and its most closely related homologs, OATs within the *SLC22* subfamily, are predicted to contain a major facilitator transporter superfamily (MFS) general fold^27^,^28^, with a secondary structure consisting of 12 transmembrane (TM) segments, a large glycosylated extracellular (EC) loop between TM1 and 2 (EC1), a large intracellular (IC) loop between TM6 and 7 (IC3), and cytoplasmic amino and carboxy termini^29^. Mutational studies and computer modelling of various members of the OAT family suggest that residues within TM1, 5, 7, 8, 10 and 11 are important for substrate recognition and activity^30^,^31^,^32^,^33^.

The rat and mouse URAT1 orthologs are functionally similar, localize to the apical membrane of kidney proximal tubule cells and share 74% amino acid identity to human URAT1 (hURAT1)^18^,^34^,^35^. However, the role of URAT1 in the mouse is unclear because knockout mice have just a slight increase in FE_UA_^36^. Also, separate studies suggest that hURAT1 differs from rat URAT1 (rURAT1) in substrate and inhibitor affinity. hURAT1 has a higher affinity for the substrate urate (*K*_m_ = 371 μM)^18^ compared to rURAT1 (*K*_m_ = 1773 μM)^35^ and also has a higher affinity for the inhibitor benzbromarone (IC_50_ = 0.13 μM)^37^ compared to rURAT1 (IC_50_ = 11.9 μM)^35^. In this study, we have confirmed this difference by directly comparing the functional activities of the human and rat transporters in the same study. Exploiting this difference in affinity between the highly related orthologs, we generated chimeras in order to map the regions of the transporter responsible for these differences. We identified several specific residues that are important in high affinity binding to hURAT1 by inhibitors and urate. Our findings suggest that two specific residues are responsible for the affinity of hURAT1 for urate, and moreover, that these same residues are responsible for the affinity of hURAT1 for all known inhibitors used in the clinic.

## Results

### TM1, 7 and 11 are important for URAT1 interaction with inhibitors

**Figure 1:**
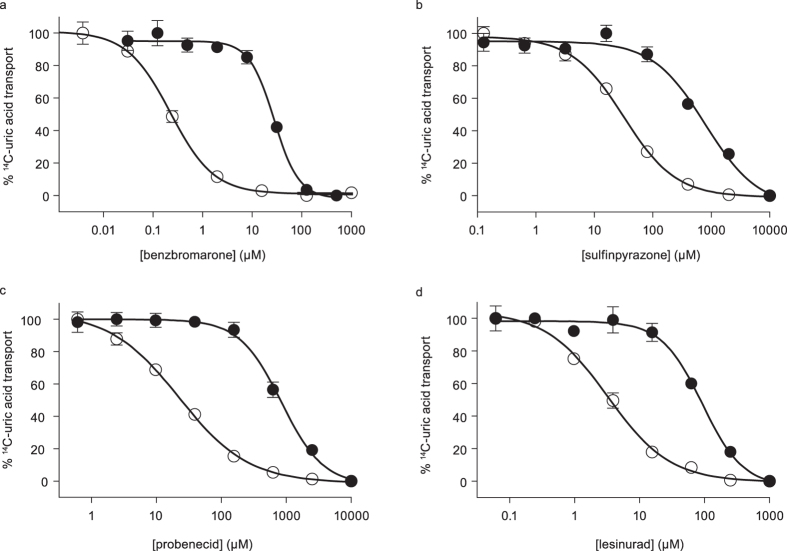
URAT1 inhibitors inhibit human URAT1 at substantially higher potency. Dose response curves of benzbromarone (**a**), sulfinpyrazone (**b**), probenecid (**c**), and lesinurad (**d**) against human URAT1 (open symbols) and rat URAT1 (closed symbols). Data are from a single experiment and are representative of multiple experiments. Points represent the mean ± standard error of the mean (SEM) from triplicate samples. The potencies (half maximal inhibitor concentration values) are shown in Supplementary Table 1. For all inhibitors, the differences in potency between human URAT1 and rat URAT1 are statistically significant (*P *< 0.0001).

Direct comparison between the human and rat URAT1 shows that hURAT1 has a significantly (*P* < 0.0001) higher affinity for the URAT1 inhibitors benzbromarone, sulfinpyrazone, probenecid, and lesinurad (Fig. 1 and Supplementary Table 2). The uric acid transport activity of human URAT1 was inhibited by benzbromarone, sulfinpyrazone, probenecid and lesinurad with half maximal inhibitory concentrations (IC_50_) of 0.22, 32, 22 and 3.5 μM, respectively, while the IC_50_ of these compounds with rat URAT1 was 26, 680, 786 and 81 μM, respectively. Therefore, compared to rURAT1, hURAT1 has a 118-fold higher affinity to benzbromarone, a 21-fold higher affinity to sulfinpyrazone, a 36-fold higher affinity to probenecid and a 23-fold higher affinity for lesinurad.

**Figure 2:**
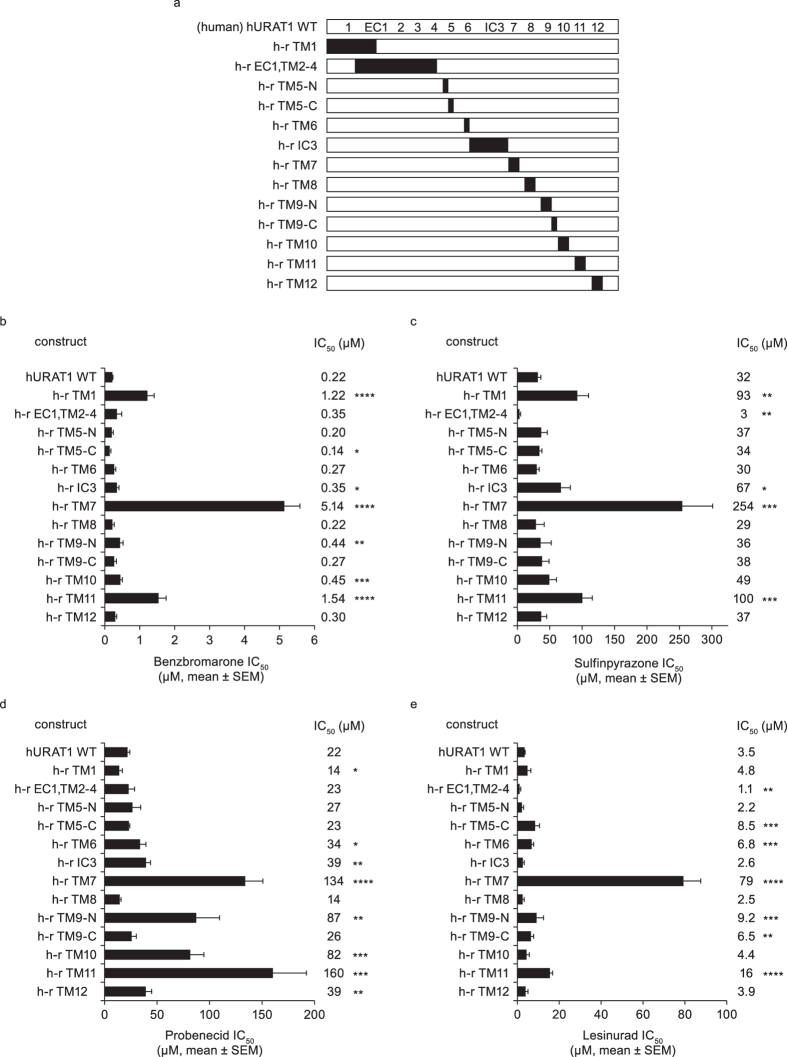
Chimeras of human (h) URAT1 and rat (r) URAT1 reveal that TM1, 7 and 11 of human (h) URAT1 confer high affinity responses to inhibitors. (**a**) Diagram of human URAT1 wild type (hURAT1 WT, white box) with the predicted secondary structures showing the approximate location of the twelve transmembrane (TM) segments (numbered), the large first extracellular loop (EC1) and the large third intracellular loop (IC3), and diagrams of individual human-to-rat (h–r) chimeras, where small regions of hURAT1 were replaced with rat URAT1 (rURAT1, black boxes). (**b–e**) Potencies against hURAT1 and the human-to-rat (h–r) chimeras shown in (**a**) for benzbromarone (**b**), sulfinpyrazone (**c**), probenecid (**d**) and lesinurad (**e**). Dose-response curves for each construct were performed as in Fig. 1, and results are the mean ± SEM from at least three experiments. Asterisks indicate a significant difference in the mean value from hURAT1 (*P < 0.05, **P < 0.01, ***P < 0.001, ****P < 0.0001).

**Figure 3:**
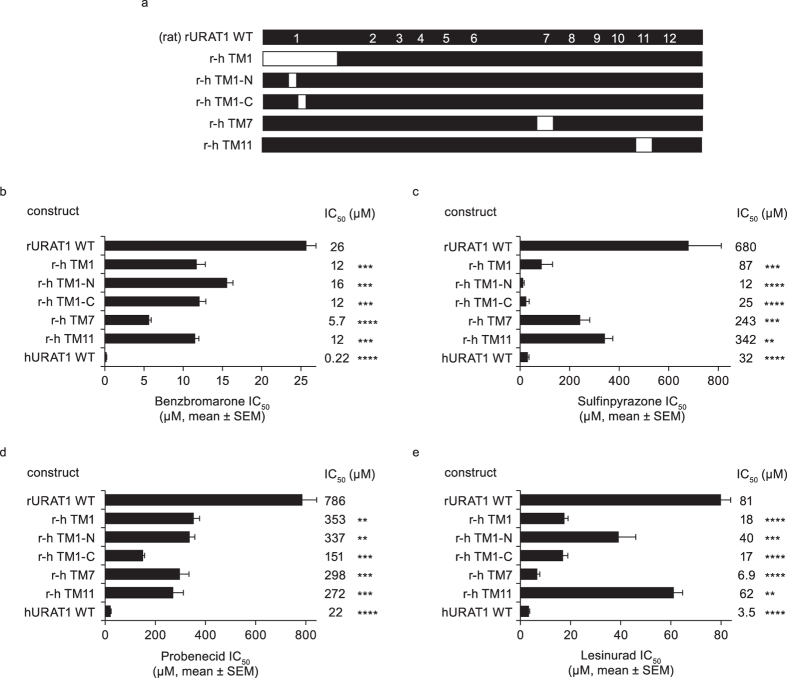
Chimeras of human (h) URAT1 and rat (r) URAT1 reveal that TMS 1, 7 and 11 of hURAT1 confer high affinity responses to inhibitors. (**a**) Diagram of rURAT1 wild type (rURAT1 WT, black box) with the predicted secondary structures showing the approximate location of the twelve transmembrane (TM) segments (numbered), the large first extracellular loop (EC1) and the large third intracellular loop (IC3), and diagrams of individual rat-to-human (r–h) chimeras where small regions of rURAT1 were replaced with human URAT1 (hURAT1, white boxes). (**b–e**) Potencies against rURAT1 and the rat-to human (r-h) chimeras shown in (**a**) for benzbromarone (**b**), sulfinpyrazone (**c**), probenecid (**d**), and lesinurad (**e**). Dose-response curves for each construct were performed as in Fig. 1, and results are the mean ± SEM from at least three experiments. Asterisks indicate a significant difference in the mean value from rURAT1 (*P < 0.05, **P < 0.01, ***P < 0.001, ****P < 0.0001).

The preferential specificity of the inhibitors for the human URAT1 ortholog suggested that amino acids present in hURAT1 and absent in rURAT1 are responsible for the high affinity interaction with URAT1 inhibitors. To identify these residues, we constructed chimeric URAT1 transporters (Figs 2a and 3a), and measured their responses to the inhibitors (Figs 2b–e and 3b–e). Specifically, we looked for decreases in affinity (loss of function) in conversions of human to rat residues, and increases in affinity (gain of function) in conversions of rat to human residues. The non-conserved residues for each chimera are listed in Supplementary Table 3, according to the alignment of the hURAT1 and rURAT1 orthologs as shown in Supplementary Fig. 1a.

hURAT1 and rURAT1 are 553 amino acid proteins with 12 transmembrane segments (TM), a single large extracellular loop (EC1), and a single large intracellular loop (IC3) (Supplementary Fig. 1a, illustrated in Figs 2a and 3a). We initially swapped the amino terminal 99 residues, a region that contains TM1 and part of EC1, and measured the inhibitory potency of URAT1 inhibitors against the uric acid transport activities. Compared to hURAT1, a chimera consisting of rURAT1 residues 1–99 and hURAT residues 100–553 (h-r TM1) had a significantly reduced affinity to benzbromarone (Fig. 2b, 6-fold reduced affinity) and sulfinpyrazone (Fig. 2c, 3-fold reduced affinity), but not to lesinurad (Fig. 2e). Paradoxically, h-r TM1 had a 1.6-fold increased affinity to probenecid (Fig. 2d). Next, we analyzed chimeras of hURAT1 that contained internal sequences of rURAT1 (Fig. 2a), scanning the remaining TM segments and IC3. With all inhibitors, significant and substantial reductions in affinity were observed with chimeras at TM7 and 11 (Fig. 2b–e, h-r TM7 and h-r TM11). Of all the chimeras, h-r TM7 had the strongest phenotype with benzbromarone (23-fold reduced affinity, Fig. 2b), sulfinpyrazone (8-fold reduced affinity, Fig. 2c), and lesinurad (23-fold reduced affinity, Fig. 2e). For probenecid, h-r TM7 had a 6-fold reduced affinity, while h-r TM11 had the strongest phenotype with a 7-fold reduced affinity (Fig. 2d). h-r TM11 also had a 7-fold, 3-fold and 5-fold reduced affinity for benzbromarone, sulfinpyrazone and lesinurad, respectively. Other chimeras also produced significant phenotypes for certain inhibitors. A chimera at TM10 (h-r TM10) had a 2-fold reduced affinity to benzbromarone and a 4-fold reduced affinity to probenecid. Some other internal chimeras (IC3 and TM12) had a significant but weaker response to probenecid, whereas a chimera containing EC loop 1 and TM2-4 (h-r EC1,TM2-4) had a paradoxically higher affinity to sulfinpyrazone.

Since the human-to-rat chimeras h-r TM1, h-r TM7, and h-r TM11 generally exhibited the strongest decreases in affinity, we focused on these regions and analyzed the corresponding rat-to-human chimeras (Fig. 3a). The chimera r-hTM1, consisting of hURAT1 residues 1–99 and rURAT1 residues 100–553, had a significantly higher affinity compared to rURAT1 to all inhibitors (Fig. 3b–e). The phenotype of this chimera suggested that hURAT1 residues within positions 1–99 confer high-affinity interactions with these URAT1 inhibitors. Within this region (residues 16–37; Supplementary Fig. 1a), we produced two rURAT1 chimeras carrying human residues within TM1 (Fig. 3a, r-h TM1-N and r-h TM1-C). Both of these chimeras displayed significantly increased affinity for all the inhibitors relative to rURAT1 (Fig. 3b–e). Strikingly, both r-h TM1-N and r-h TM1-C had a lower affinity to sulfinpyrazone than human URAT1 (Fig. 3c). Rat chimeras containing human TM7 (r-h TM7) and human TM11 (r-h TM11) also showed increased affinity for inhibitors compared to rat URAT1 (Fig. 3b–e). In summary, rat-to-human chimeras within TM1, 7 and 11 significantly increased affinity for all inhibitors, consistent with the reduced affinity for the corresponding human-to-rat chimeras. The results suggested that residues within these transmembrane segments are important in the response to URAT1 inhibitors.

### hURAT1 serine-35, phenylalanine-365 and isoleucine-481 mediate high affinity interactions with URAT1 inhibitors

**Figure 4:**
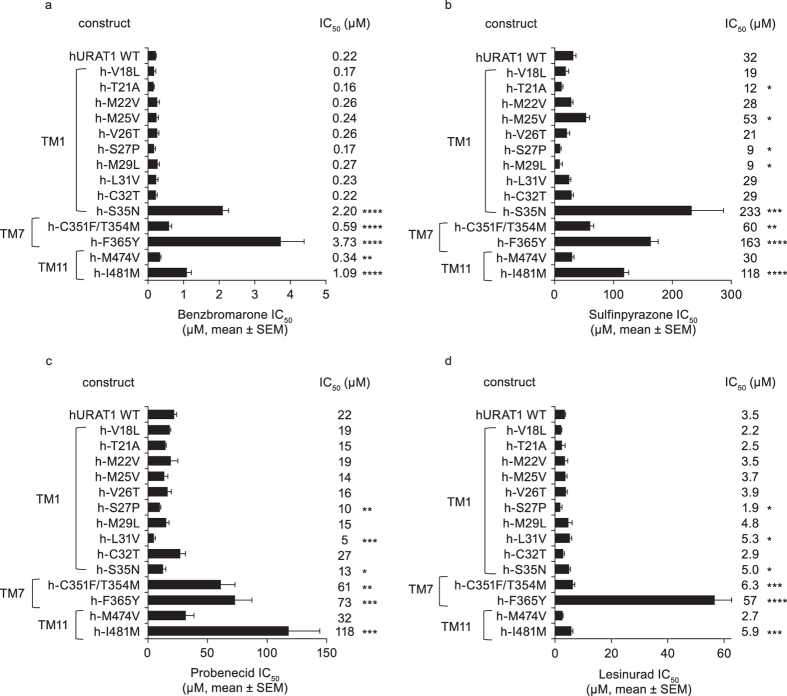
Identification of individual residues in TM1, 7 and 11 of hURAT1 that confer high affinity interaction with inhibitors: analysis of human-to-rat point mutants. Potency (IC_50_) of URAT1 inhibitors benzbromarone (**a**), sulfinpyrazone (**b**), probenecid (**c**) and lesinurad (**d**) against human URAT1 wild type (hURAT1 WT) and point mutant chimeras with rat URAT1 (rURAT1) residues in TM1, 7, and 11. Dose-response curves were performed as in Fig. 1, and results are the mean ± SEM from at least three experiments. In general, hURAT1 residues Ser-35, Phe-365 and Ile-481 are important for high affinity interaction with URAT1 inhibitors. Asterisks indicate a significant difference in the mean value from hURAT1 WT (*P < 0.05, **P < 0.01, ***P < 0.001, ****P < 0.0001).

**Figure 5:**
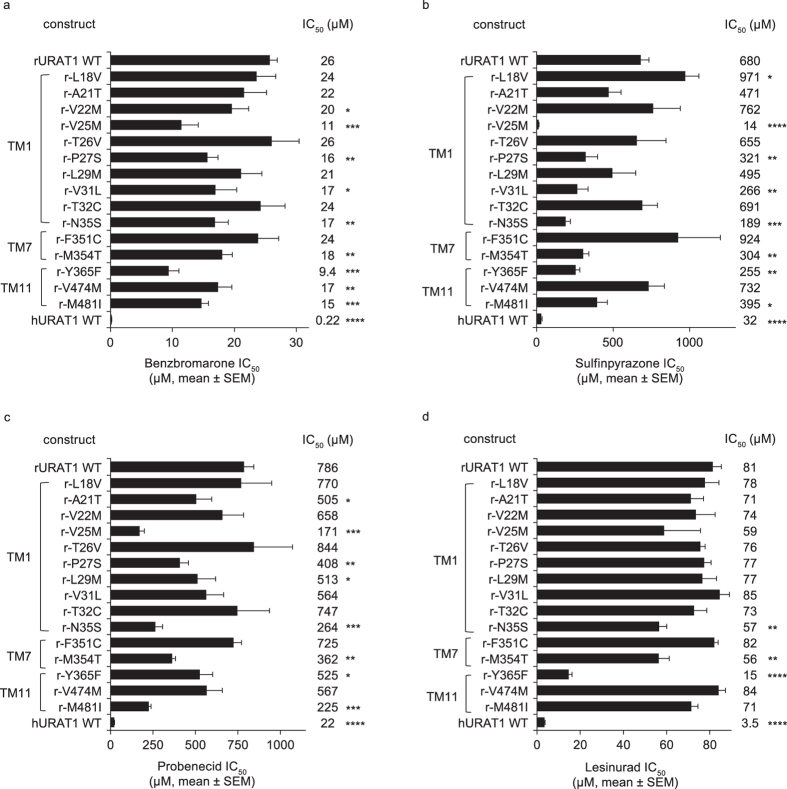
Identification of individual residues in TM1, 7 and 11 of hURAT1 that confer high affinity interaction with inhibitors: analysis of rat-to-human point mutants. Potency (IC_50_) of URAT1 inhibitors benzbromarone (**a**), sulfinpyrazone (**b**), probenecid (**c**) and lesinurad (**d**) against rat URAT1 wild type (rURAT1 WT) and point mutant chimeras with human URAT1 (hURAT1) residues in TM1, 7 and 11. Dose-response curves were performed as in Fig. 1, and results are the mean ± SEM from at least three experiments. In general, hURAT1 residues Ser-35, Phe-365 and Ile-481 are important for high affinity interaction with URAT1 inhibitors. Asterisks indicate a significant difference in the mean value from rat URAT1 wild type (WT) (*P < 0.05, **P < 0.01, ***P < 0.001, ****P < 0.0001).

To identify specific residues within TM1, 7 and 11 that are involved in the interaction with URAT1 inhibitors, we produced point mutant chimeras for all non-conserved residues within these regions (Supplementary Fig. 1a and Supplementary Table 3) and then analyzed their inhibitor responses (Figs 4 and 5 and Supplementary Table 2). The expression of many of these point mutant chimeras was also analyzed by Western blotting (Supplementary Figure 2), showing that they are equivalently expressed relative to the wild type transporters. A point mutant chimera of hURAT1 carrying rat Tyr-365 (h-F365Y) had a strong phenotype with all inhibitors. Compared to hURAT1, h-F365Y had reduced affinities of 17-fold, 5-fold, 3-fold and 16-fold to benzbromarone (Fig. 4a), sulfinpyrazone (Fig. 4b), probenecid (Fig. 4c) and lesinurad (Fig. 4d), respectively. The strong phenotype of h-F365Y demonstrates the importance of human URAT1 Phe-365, located in TM7, for inhibitor potency, and is consistent with the phenotype of the chimera h-r TM7. WithinTM7, residues 351 and 354 also differ between hURAT1 and rURAT1 (Supplementary Fig. 1a and Supplementary Table 2). The double point mutant chimera of hURAT1 carrying these two rat residues, h-C351F/T354M, also showed a significant, albeit weaker phenotype, with the inhibitors (Fig. 4 and Supplementary Table 2). Therefore, Cys-351 and Tyr-354 have a minor role, while Phe-365 is the most important residue in TM7 of human URAT1 that influences inhibitor potency.

From residues in TM1, a point mutant of hURAT1 carrying rat Asn-35 (h-S35N) produced the strongest phenotype (Supplementary Table 2). h-S35N had a 10-fold, 7-fold and 1.4-fold reduced sensitivity to benzbromarone (Fig. 4a), sulfinpyrazone (Fig. 4b) and lesinurad (Fig. 4d), respectively, demonstrating the importance of hURAT1 Ser-35 for inhibitor potency, which is consistent with the phenotype of the chimera h-r TM1. Paradoxically, h-S35N had a significant 1.7-fold increased potency with probenecid, and other point mutants in TM1 also have increased affinity (h-S27P and h-L31V) for this inhibitor. However, these phenotypes for probenecid are consistent with the phenotype of h-r TM1. For sulfinpyrazone (Fig. 4b), a point mutant chimera of hURAT1 carrying rat Val-25 (h-M25V) had a 1.7-fold reduced affinity, indicating a role for hURAT1 Met-25 that was further revealed with the converse rat URAT1 point mutant r-V25M (below, Fig. 5). In general, for the human URAT1 point mutants carrying individual rat residues in TM1, hURAT1 Ser-35 is the most important residue in TM1 for inhibitor affinity.

In TM11, a point mutant of hURAT1 carrying rat Met-481 (h-I481M) had a 5-fold, 4-fold, 5-fold and 2-fold reduced affinity to benzbromarone (Fig. 4a), sulfinpyrazone (Fig. 4b), probenecid (Fig. 4c) and lesinurad (Fig. 4d). This phenotype is consistent with the phenotype of the TM11 chimera h-r TM11. The only other non-conserved residue in TM11 is Met-474 (Supplementary Fig. 1a and Supplementary Table 3), and the point mutant chimera at this position (h-M474V) had a weak phenotype for benzbromarone only. Therefore, in TM11, human URAT1 Ile-481 is important for interactions with all inhibitors.

The human-to-rat point mutant chimeras in TM1, 7 and 11 highlight the importance of human URAT Ser-35, Phe-365 and Ile-481 for URAT1 inhibitor affinity. Both Phe-365 and Ile-481 are important for interactions with all the tested inhibitors, with Phe-365 generally showing the strongest phenotype. In addition, Ser-35 produced a strong phenotype for benzbromarone and sulfinpyrazone, but not for the other inhibitors, suggesting subtle but significant differences between the interactions of each inhibitor with the binding site in URAT1. The involvement of additional residues (such as Met-25 in TM1) was also revealed from these analyses.

We next looked for gain of function mutations in rURAT1 carrying the corresponding hURAT1 residues in TM1, 7 and 11 (Fig. 5). Compared to rURAT1, the point mutant with hURAT1 Phe-365, r-Y365F, had a significant increase in affinity for all inhibitors. It had the strongest phenotype for all the point mutants with benzbromarone (Fig. 5a) and lesinurad (Fig. 5d), a 2.8- and 5.4-fold increase in affinity, respectively. This point mutant also had a 2.7-fold increased affinity for sulfinpyrazone (Fig. 5b) and a 1.5-fold increased affinity for probenecid (Fig. 5c). Point mutants in other residues of TM7, r-F351C and r-M354T, had either a weaker or no phenotype compared to r-Y365F, with the exception of r-M354T that had a stronger phenotype for probenecid. The gain-of-function results for r-Y365F provide strong evidence for a direct interaction between Phe-365 and the inhibitors.

The rURAT1 point mutant carrying hURAT1 Ser-35 in TM1, r-N35S, also had a significant gain of function phenotype for all inhibitors. This increase in affinity was 1.5-fold for benzbromarone, 3.6-fold for sulfinpyrazone, 3.0-fold for probenecid and 1.4-fold for lesinurad. However, except for lesinurad, the TM1 point mutant with the strongest phenotype is at position 25. rURAT1 carrying hURAT1 Met-25, r-V25M, had a 2.4-, 49- and 4.6-fold increased affinity for benzbromarone, sulfinpyrazone and probenecid, respectively. The increase in affinity of this single point mutant to sulfinpyrazone is striking, and it had a higher affinity to this inhibitor than hURAT1 (Fig. 5b). The rURAT1 point mutant carrying hURAT1 Ser-27 had the same trend as r-V25M but with a weaker phenotype. r-P27S had a 1.6-, 2.1- and 1.9-fold higher affinity to benzbromarone, sulfinpyrazone and probenecid, respectively, and no phenotype to lesinurad. Generally, the phenotypes of these point mutants are consistent with the phenotypes of r-h TM1-N and r-h TM1C from Fig. 3, and reveal the importance of TM1 residues 25, 27 and 35.

In TM11, the rURAT1 point mutant carrying hURAT1 Ile-481, r-M481I, had a significant 1.7-, 1.7- and 3.5-fold increased affinity to benzbromarone, sulfinpyrazone and probenecid, respectively. It had no phenotype for lesinurad. Meanwhile, rURAT1 carrying hURAT1 Met-474, r-V474M, had 1.5-fold increased affinity for benzbromarone and no phenotype for the other inhibitors. Therefore, in TM11, hURAT1 Ile-481 contributes to inhibitor affinity.

Consistent with the results for the human to rat point mutants, the findings for the rat-to-human point mutants also highlight the importance of human residues Ser-35 in TM1, Phe-365 in TM7 and Ile-481 in TM11 for inhibitor affinity. Additionally, the rat-to-human point mutants at residues 25 and 27 in TM1 demonstrate a role for Met-25 and Ser-27 for inhibitor interactions.

### A three-dimensional model of OAT1, a homolog of URAT1, predicts that Ser-35, Phe-365 and Ile-481 are in close proximity

**Figure 6:**
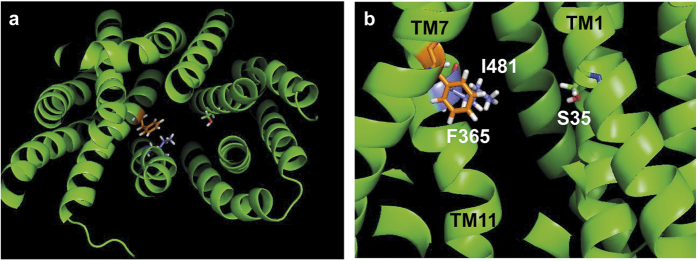
Human URAT1 residues Ser-35, Phe-365 and Ile-481 are likely in close proximity and the sidechains project into the central transporter channel. Pymol (Schrödinger) images of human OAT1 (hOAT1) three-dimensional model^33^, a homolog of human URAT1 (hURAT1), highlighting Ser-35 (S35, multi-colored), Phe-365 (F365, orange), and Ile-481 (I481, purple). hOAT1 residues Asn-35 and Tyr-354 were converted to the corresponding URAT1 residues Ser-35 and Phe-365, while conserved hOAT1 residue Ile-470 corresponds to hURAT1 Ile-481. (**a**) End view with the cytoplasmic surface facing the viewer. (**b**) Side view with extracellular surface facing up. For both panels, the front part of the protein has been removed to reveal the central pore.

Using the crystal structure of the bacterial glycerol-3-phosphate transporter^27^, a three-dimensional model of human OAT1, a homolog of hURAT1, was developed and tested^33^. Human OAT1 transports various organic anions including urate^38^, and has a 46% amino acid identity to hURAT1 (Supplementary Fig. 1b). The hURAT1 residues Ser-35, Phe-365 and Ile-481 correspond to human OAT1 residues Asn-35, Tyr-354 and Ile-470 (Supplementary Fig. 1b). Remarkably, in the human OAT1 three-dimensional model, Ser-35, Phe-365 and Ile-481, the residues identified in the unbiased functional mapping procedure, are predicted to be in close proximity and project within the central channel of the protein (Fig. 6a,b). TM7 and 11 come together so that the sidechains of Phe-365 (F365) and Ile-481 (I481) are adjacent to one another. Ser-35 (S35) occurs on the opposite side of the channel, yet its sidechain is close to the sidechains of the other residues. In a side-view of the protein (Fig. 6b), all three residues line up on a plane that is perpendicular to the membrane. Confirmation of this prediction will require crystallization of the protein, however, this three-dimensional model suggests that these residues form a URAT1 inhibitor binding site, supporting the mapping data that each is important for inhibitor function.

**Figure 7:**
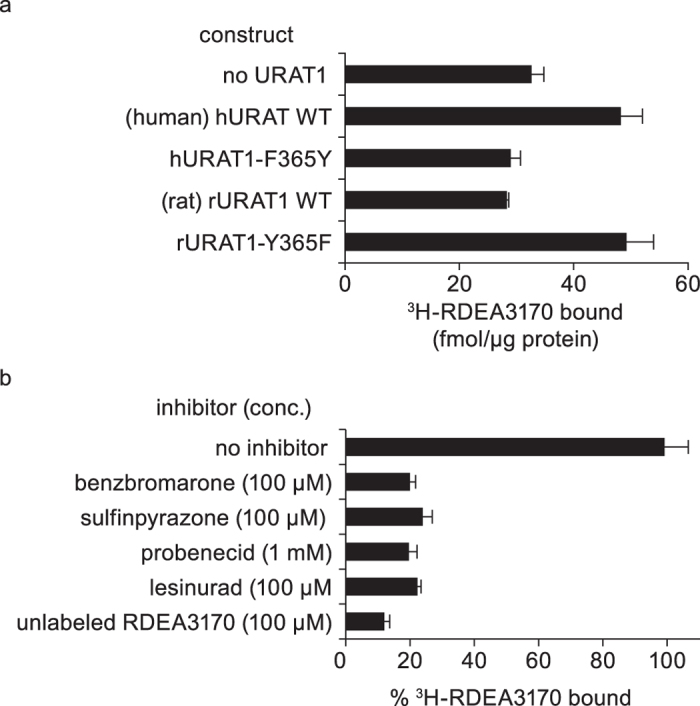
In a novel human URAT1 binding assay that requires Phe-365, inhibitors displace the binding of the probe. (**a**) ^3^H-RDEA3170 binds to human URAT1 and not rat URAT1, and binding depends on Phe-365. Binding was assayed after incubation of 500 nM ^3^H-RDEA3170 with membranes prepared from cells transfected with the indicated constructs. (**b**) Inhibitors block binding of ^3^H-RDEA3170 to human URAT1. Human URAT1 membranes were incubated with 10 nM of ^3^H-RDEA3170 in the absence or presence of the indicated concentrations of inhibitors. Results are the mean ± SEM from samples assayed in triplicate.

The mapping data coupled with the model suggest that all clinical uricosuric agents bind directly to the same site within URAT1. We tested this notion in a novel direct binding assay using ^3^H-RDEA3170, a radiolabeled high affinity (*K*_m_ = 20 nM) URAT1 inhibitor probe^39^. ^3^H-RDEA3170 is also dependent on hURAT1 Phe-365 for binding (Fig. 7a). Moreover, benzbromarone, sulfinpyrazone, probenecid and lesinurad all displace binding of the probe to hURAT1 protein (Fig. 7b), further suggesting that the inhibitors interact at the same URAT1 binding site.

### Human URAT1 Ser-35, Phe-365 and Ile-481 act additively to enhance affinity to URAT1 inhibitors

**Figure 8:**
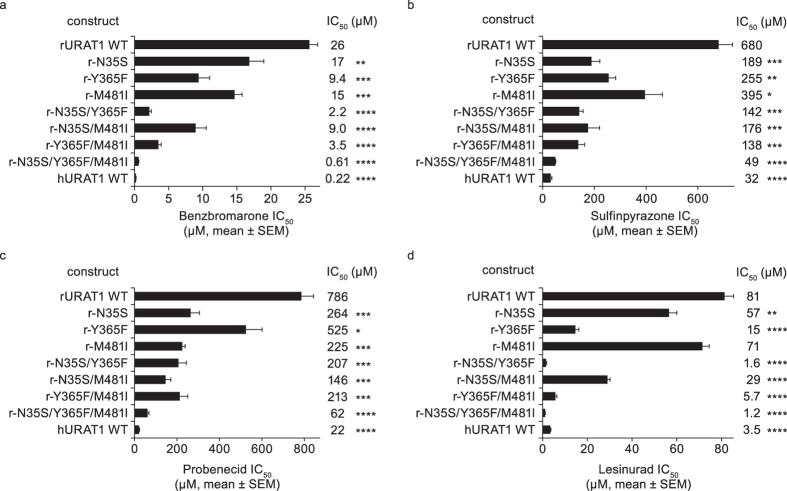
Human URAT1 residues 35, 365, and 481 act additively to enhance affinity to URAT1 inhibitors. Potencies of benzbromarone (**a**), sulfinpyrazone (**b**), probenecid (**c**), and lesinurad (**d**) against rat URAT1 wild type (rURAT1 WT) and individual or combination point mutants with human URAT1 (hURAT1) residues at positions 35, 365, and 481. Dose-response curves were performed as in Fig. 1, and results are the mean ± SEM from at least three experiments. Asterisks indicate a significant difference in the mean value from rURAT1 WT (*P < 0.05, **P < 0.01, ***P < 0.001, ****P < 0.0001).

The OAT1 computer model suggested that hURAT1 Ser-35, Phe-365 and Ile-481 can act additively to confer high affinity inhibitor interactions. Therefore, to test this hypothesis, we produced rURAT1 point mutants with combinations of the individual hURAT1 residues and tested their response to URAT1 inhibitors. We found that combinations of the point mutants produce phenotypes that are additive, with the double point mutants showing higher affinity for all of the URAT1 inhibitors compared with the individual single point mutants (Fig. 8). For example, with benzbromarone (Fig. 8a), the double point mutant at positions 35 and 365 (r-N35S/Y365F) had an increased affinity of 12-fold, a stronger phenotype compared to the single point mutants r-N35S and r-Y365F. The additional point mutation at position 481, the triple point mutant r-N35S/Y365F/M481I, had an even stronger phenotype with an increased affinity of 42-fold. These trends were similar for the other URAT1 inhibitors (Fig. 8b–d). The triple point mutant rURAT1-N35S/Y365F/M481I showed the highest affinity for all the URAT1 inhibitors, and the affinity of this construct is quite similar to that of hURAT1 for inhibitors (Fig. 8a–d). These findings suggest that these three residues functionally cooperate to interact with URAT1 inhibitors.

### Substrate interactions are also associated with this region of hURAT1

**Figure 9:**
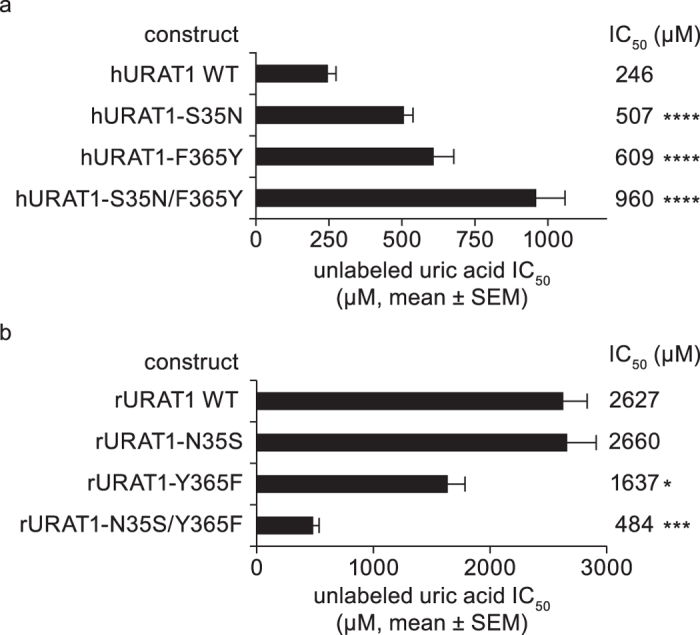
Human URAT1 Ser-35 and Phe-365 confer high affinity interaction with urate. Potencies of uric acid against wild type (WT) and chimeric point mutants for human URAT1 (hURAT1, **a**) and rat URAT1 (rat URAT1, **b**) are shown. Dose-response curves were performed as in Fig. 1 using unlabelled uric acid, and results are the mean ± SEM from at least three experiments. Asterisks indicate a significant difference in the mean value of the sample from wild type (*P < 0.05, ***P < 0.001, ****P < 0.0001). For the mean IC_50_ difference between hURAT1 and rURAT1, *P* < 0.0001.

We tested whether the hURAT1 residues identified using inhibitors are also important for interactions with the URAT1 substrate uric acid. Similar to inhibitors, previous studies have suggested that hURAT1 has a higher affinity for uric acid relative to rat URAT1^18^,^35^. We confirmed this result in a competition study using unlabelled uric acid. Indeed, unlabelled uric acid inhibits the transport of radiolabeled uric acid at a greater than 10-fold higher potency for hURAT1 (IC_50_ = 246 μM, Fig. 9a) than for rat URAT1 (IC_50_ = 2627 μM, Fig. 9b) (*P* < 0.0001). We then tested key residues within human and rat transporters for a role in uric acid affinity. Single point mutant chimeras of hURAT1 carrying rat Asn-35 and Phe-365, h-S35N and h–F365Y, both showed a significant 2.1- and 2.5-fold decrease in affinity to uric acid (loss of function), respectively, relative to hURAT1, and the double point mutant h–S35N/F365Y showed a greater additive 3.9-fold decrease in affinity to uric acid (Fig. 9a). For the converse chimeras, rURAT1 with hURAT1 Ser-35, r-N35S, had no phenotype, whereas r–Y365F had significant 1.6-fold increased affinity to uric acid (gain of function) relative to rat URAT1 (Fig. 9b). Interestingly, the double point mutant chimera rURAT1-N35S/Y365F exhibited a 5.4-fold increase in affinity for uric acid (Fig. 9b), approaching the affinity of hURAT1. Thus, Ser-35 and Phe-365 in hURAT1 are important and act additively in substrate affinity, similar to their role in URAT1 inhibitor affinity. These findings are consistent with saturation transport kinetics experiments measuring the affinity (*K*_m_) for urate^40^. It is therefore highly likely that inhibitors block URAT1 function by preventing substrate interaction within the central portion of the channel.

## Discussion

In this study, we exploited differences between human and rat URAT1 to reveal molecular interactions with the substrate urate and with clinically relevant URAT1 inhibitors, compounds that reduce sUA levels for the treatment of gout. Using hURAT1 and rURAT1 chimeras, we were able to identify hURAT1 residues that confer high affinity interaction with URAT1 inhibitors. Residues responsible for the high affinity of hURAT1 to inhibitors include Ser-35 in TM1, Phe-365 in TM7 and Ile-481 in TM11, which correspond to Asn, Tyr and Met, respectively, in rURAT1. For urate, hURAT1 Ser-35 and Phe-365 mediate high affinity interaction. Urate and inhibitors interact with a common binding site on URAT1 because all require similar URAT1 residues for high affinity interaction. The point mutants in rURAT1 that carry the individual hURAT1 residues Ser-35, Phe-365 and Ile-481 all increase the potency of the inhibitors. These ‘gain of function’ phenotypes provide the strongest evidence that these human URAT1 residues are important for interactions with both inhibitors and substrate.

Human URAT1 Ser-35, Phe-365, and Ile-481 were identified in an unbiased screen for URAT1 amino acids involved in affinity for inhibitors. These three amino acids co-localize in the central channel of a computer model of human OAT1^33^, a homolog of hURAT1 that also transports urate^38^ and is inhibited by probenecid^41^. Furthermore, chimeric point mutant combinations of these residues produced additive phenotypes for affinity to inhibitors and urate, suggesting they form a common binding site for URAT1 substrates and inhibitors within the transporter channel. This notion is supported by recent findings from the crystal structures of the GLUT glucose transporters^42^,^43^,^44^, SLC family members that have weak sequence homology but are structurally related to URAT1 through the MFS fold^45^. These studies show that amino acid sidechains in TM1, TM7 and TM11 (as well as residues in other TM segments) directly contact substrates for the GLUTs. Furthermore, in the inward-open conformation of GLUT1 and GLUT5, the extracellular sides of TM1 and TM7 contact each other^42^, consistent with the predicted close proximity of URAT1 Ser-35 and Phe-365 in the OAT1 computer model, which is in the same conformation. In another computer model of OAT1, simulated conformational changes were observed in the extracellular sides of TM1 and TM7^46^, suggesting a possible role for substrate transport for residues within these domains. The precise positioning of these URAT1 residues within the protein is speculative and awaits clarification through determination of a URAT1 crystal structure.

We predict that these URAT1 residues directly contact both substrates and inhibitors. In support of this hypothesis, we developed a novel and specific human URAT1 binding assay. All inhibitors displaced binding of the radiolabeled probe, suggesting that our functional mapping data uncovered a specific binding site within URAT1. This binding assay offers a new tool for further characterizing the molecular interactions of compounds and substrates to URAT1. All uricosuric agents we have tested to date interact with these amino acids within URAT1. The inhibitors themselves were not identified and developed through standard medicinal chemistry approaches and so are structurally very diverse. We believe the commonality of binding may be due to the unique properties of the residues in this region facilitating inhibitor interactions.

Although the precise nature of these interactions is unknown, rURAT1 may have a lower affinity for urate and the inhibitors because the residues at positions 35, 365 and 481 are bulkier than the corresponding hURAT1 residues; therefore, steric hindrance may reduce the affinity of interactions with the rat transporter. Residue 365 occurs in a cluster of aromatic residues in TM7 that are highly conserved in the SLC22 transporter family, a domain shown to be important in substrate interactions for OAT1 and OAT3^29^,^30^. However, unlike URAT1 in which both Phe-365 and Tyr-365 support transport activity, the corresponding residues in human OAT1 and rat OAT3, Tyr-354 and Tyr-352, are strictly required for substrate recognition. Mutations to phenylalanine are inactive^29^,^30^, showing that substrate recognition occurs through hydrophilic contacts with the tyrosine hydroxyl groups. It therefore appears that recognition of urate and URAT1 inhibitors through residue 365 is mechanistically different, possibly occurring through hydrophobic interactions between aromatic moieties. The hydroxyl group of Tyr-365 of rURAT1 may sterically hinder this hydrophobic interaction to reduce affinity.

Previously, we reported that Phe-365 and Met-25 were acquired during the evolution of simians (humans, apes, Old World monkeys, and New World monkeys), and that these residues promote higher urate affinity in simian URAT1, relative to non-simian URAT1, which carry Tyr-365 and Val-25^40^ (Supplementary Table 4). Residues 35 and 481 have distinct phylogenetic distributions (Supplementary Table 4) but also differ between human, rat, and mouse, and so were also identified in analyses of human-to-rat URAT1 chimeras. We expect that other residues are also involved in urate and inhibitor affinity. Because hURAT1 and rURAT1 share a 74% amino acid identity, chimeras from these orthologs will not identify all residues involved in inhibitor binding. Based on structure/function analysis of URAT1 homologs^31^,^33^,^45^,^46^,^47^,^48^,^49^ as well as from recent findings from GLUT crystal structures^41^,^44^, we expect that conserved residues in many TM segments also play a role in binding to URAT1 substrates and inhibitors. The identity of residues corresponding to human URAT1 residues 35, 365, and 481 in other URAT1 species (orthologs) and in *SLC22A* subfamily homologs is shown in Supplementary Table 4. Interestingly, a tyrosine residue occurs in most homologs at the position corresponding to hURAT1 residue 365, so that Phe-365 is nearly unique to hURAT1. Therefore, this phenylalanine may be important in the high potency and specificity of benzbromarone and verinurad for hURAT1 (Tan *et al*., manuscripts submitted). However, probenecid is more non-specific and has a similar potency to hURAT1, hOAT4, hOAT1, and hOAT3^24^ consistent with a finding that URAT1 residues 35, 365, and 481 all occur within sequence motifs common to all SLC22A family members^49^.

In summary, we have identified several amino acids in hURAT1 that mediate the high affinity interaction with URAT1 inhibitors. Some of these residues also participate in the recognition and affinity for the URAT1 substrate uric acid. This provides a facile mechanism for inhibition of URAT1: inhibitors sterically hinder the interaction of urate with key amino acids within the central channel of URAT1 to prevent uric acid transport. Naturally occurring polymorphisms in these amino acids could in principle impact the efficacy of URAT1 inhibitors, though none have been identified to date. These results could also assist in the discovery of new high affinity and specific inhibitors of URAT1, which may also serve as safer and more effective urate-lowering therapies for hyperuricemia and gout.

## Materials and Methods

### Compounds and substrates

Benzbromarone and sulfinpyrazone were obtained from Sigma-Aldrich. Lesinurad, 2-((5-bromo-4-(4-cyclopropylnaphthalen-1-yl)-4H-1,2,4-triazol-3-yl)thio)acetic acid, was synthesized at Ardea Biosciences. These URAT1 inhibitors were diluted in 20 or 100 mM DMSO stock solutions. Water-soluble probenecid (Life Technologies) was prepared according to the manufacturer’s instructions. ^14^C-uric acid (50–60 mCi/mmol, 0.5 mCi/ml), was from American Radiolabeled Chemicals, Inc. ^3^H-RDEA3170, 2-((3-(4-cyanonaphthalen-1-yl)pyrindin-4-yl)thio)-2-methylpropanoic acid^39^, was synthesized by Moravek Biochemicals with a specific activity of 21.3 Ci/mmol and a concentration of 1 mCi/ml, at a purity of 99%, with tritiated methyl groups.

### Constructs and mutagenesis

hURAT1 (GenBank BC053348.1) and rURAT1 (NCBI NM_001034943.1) genes were purchased from Origene Technologies, Inc. and subcloned into pCMV6/neo using *Not*I, creating pCMV6/neo-hURAT1 and pCMV6/neo-rURAT1. Mutants were produced by polymerase chain reaction (PCR) or site-directed mutagenesis using the QuikChange Lightning Multi Site-Directed Mutagenesis Kit (Agilent Technologies). All mutants were confirmed by DNA sequencing. Detailed methods are provided in Supplementary Methods, and primers are listed in Supplementary Table 1. All reported mutants displayed urate transport activity. Some other mutants were inactive (data not shown). Residues in rURAT1 and hOAT1 that correspond to the residues in hURAT1 were identified from aligned protein sequences (Supplementary Fig. 1)^50^.

### Cell culture and transfection

HEK-293T cells were maintained in DMEM/high glucose/L-glutamine/HEPES supplemented with 1 mM sodium pyruvate (Life Technologies) and 10% FBS (PAA Laboratories) at 37 °C in 5% CO_2_. To express URAT1, plasmids were reverse transfected into HEK-293T cells. DNA (10 μg) was mixed with 30 μl of DreamFect Gold (Boca Scientific) in 2 ml OptiMem (Life Technologies). After 20 minutes, 18 ml of media containing 2 × 10^7^ cells in suspension was added and mixed, and 200 μl per well were plated onto white clear-bottomed poly-D-lysine coated 96-well plates (BD Biosciences). The cells were assayed the following day.

### Transporter activity assays

With the URAT1 inhibitors, activity assays were performed in assay buffer consisting of 25 mM HEPES (from a 1 M solution at pH 7.3; USB Corporation), 125 mM sodium gluconate, 4.8 mM potassium gluconate, 1.2 mM monobasic potassium phosphate, 1.2 mM magnesium sulfate, 1.3 mM calcium gluconate and 5.6 mM glucose. Compounds were serially diluted into assay buffer and added to the cells for 5 minutes prior to addition of radiolabelled uric acid substrate. URAT1-expressing cells were incubated with 100 μM ^14^C-uric acid for 10 minutes. Cells were then washed three times in 25 mM HEPES/125 mM sodium gluconate (wash buffer), and solubilized in Ultima Gold (Perkin Elmer) prior to liquid scintillation counting. Each treatment was measured in triplicate.

Activity assays using lesinurad and unlabelled uric acid were performed in a similar way, except that the HEPES in the assay and wash buffers was replaced by 25 mM MES, from a 1 M MES solution (Sigma) adjusted to pH 5.5 with sodium hydroxide. Unlabelled uric acid was prepared at a 20 mM stock just prior to use in a solution containing 20 mM dibasic potassium phosphate and 20 mM potassium hydroxide.

Nonlinear regression analyses using GraphPad Prism software were used to calculate the potency of URAT1 inhibitors (IC_50_ values) from log (inhibitor) versus response - variable slope (four parameters) equations. Statistical significance was assessed using Student’s unpaired t-tests.

### Binding assays

Membranes were prepared from URAT1-transfected cells. Cells (5–10 × 10^8^) were harvested in 1 ml ice-cold binding buffer (25 mM HEPES pH 7.3, 125 mM sodium gluconate) containing complete, EDTA-free protease inhibitor cocktail (Sigma). Cells were lysed with 100 strokes in a dounce homogenizer using a tight-fitting “B” stem. Lysates were transferred to 1.5 ml Eppendorf tubes and centrifuged at 250 rcf for 5 minutes at 2 °C, and supernatants were transferred to fresh tubes and centrifuged again at 18,000 rcf for 20 minutes at 2 °C. The pellets (membrane fractions) were resuspended in 1 ml of ice-cold binding buffer containing protease inhibitors and frozen at −80 °C. Protein was measured using the Bio-Rad protein assay dye reagent concentrate.

To initiate binding, membranes at 2.5 μg total protein were incubated with ^3^H-RDEA3170 for 30 minutes at room temperature. Samples were then subjected to rapid filtration through 25 mm glass fibre/1.2 μM PES 96-well filter plates (Corning FiltrEX 3510), and washed once with 200 μl ice-cold binding buffer. The plates were then subjected to scintillation counting. All samples were assayed in triplicate.

## Additional Information

**How to cite this article**: Tan, P. K. *et al*. Mechanism of high affinity inhibition of the human urate transporter URAT1. *Sci. Rep*. **6**, 34995; doi: 10.1038/srep34995 (2016).

## Supplementary Material

Supplementary Information
