# Supplementary material for: Mechanism of high affinity inhibition of the human urate transporter URAT1
Source: Sci Rep. 2016 Oct 7;6:34995. doi: 10.1038/srep34995 (PMC5054527; doi:10.1038/srep34995)
Supplement: Supplementary Information [file srep34995-s1.pdf]

Mechanism of high affinity inhibition of the human urate transporter URAT1

Philip K. Tan*, Traci M. Ostertag, and Jeffrey N. Miner

Department of Biology, Ardea Biosciences (A member of the AstraZeneca Group), 9390 Towne Centre Drive, San Diego, CA, 92121 USA

Corresponding Author:

*Philip K. Tan

Address: Ardea Biosciences, 9390 Towne Centre Drive, San Diego, CA 92121

Phone: (858) 652-6591. Fax: (858) 625-0760. Email ptan@ardeabio.com

SUPPLEMENTARY INFORMATION

Supplementary methods

Construction of chimeras. All chimeras were produced using mutagenic primers listed in Table S1. Some primers were designed to introduce new restriction enzyme sites without altering the coded amino acid sequence (silent mutagenesis), and in this way the same restriction enzyme sites were introduced at equivalent positions of both hURAT1 and rURAT1. For example, the primers h-HindIII and h-NheI-2 were used to introduce *Hind*III and *Nhe*I sites, respectively, into hURAT1, and the primers r-HindIII and r-NheI-2 were used to introduce the same sites into the equivalent positions of rURAT1. Digestion of these constructs with *Hind*III-*Nhe*I, mixing of the large hURAT1 fragment with the small rURAT1 fragment, and ligation produced h-r TM8 (hURAT1 with rURAT1 residues at TM8). Using this method, the following chimeras were produced and analyzed in this study: h-r EC1, TM2-4, h-r IC3, h-r TM7, h-r TM8, h-r TM9 and r-h TM7. For producing chimeras in TM7, the existing *Bsp*EI site in hURAT1 was used. Other chimeras were produced directly from single mutagenic primers.

Construction of h-r TM1 and r-h TM1 chimeras. Primers int F and int R were engineered to hybridize to both hURAT1 and rURAT1 nucleotides 268-291, for construction of chimeras using a sequential two-step PCR and subcloning. In the first PCR reactions, hURAT1 and rURAT1segments were amplified from pCMV6/neo-hURAT1 and pCMV6/neo-rURAT1 using primer pairs 5'-ext F/ int R and int F/3'-ext R. The products from the first PCR reactions were then mixed together, along with the primer pair 5'-ext F and 3'-ext R for a second round of PCR reactions to produce the chimeric genes. These PCR products were then digested with *Not*I and subcloned into pCMV6/neo. Chimeric clones were then identified by restriction enzyme analysis and verified by sequencing. Two chimeras were produced: the first that is hURAT1 from amino acids 1-99 and rURAT1 from amino acids 100-553 (hr99) and the second that is rURAT1 from amino acids 1-99 and hURAT1 from amino acids 100-553 (rh99).

Western blotting. The procedure for Western blotting of URAT1 constructs is described in reference 39.

SUPPLEMENTARY FIGURE LEGENDS

Supplementary Figure 1. Alignment of protein sequences of human URAT1 and rat URAT1 (a) and human URAT1 and human OAT1 (b). Sequences from UniProt[46] (human URAT1, Q96S37; rat URAT1, Q3ZAV1 and human OAT1, Q4U2R8) were aligned using NCBI protein BLAST[36]. The locations of the transmembrane (TM) segments, indicated in green boxes, was obtained from UniProt and also inferred from the location of intracellular signature sequences that are conserved with related transporters (red underlines)[47]. The locations of the large extracellular loop (EC1) and large intracellular loop (IC loop 3) are also shown.

Supplementary Figure 2. Western blot analysis of expression of hURAT1, rURAT1, and point mutant chimeras. Cells were transfected with constructs expressing untagged human

(h) URAT1, HA-tagged hURAT1 or HA-tagged rat (r) URAT1, and membrane extracts from transfected cells were probed with antibodies against HA and β-actin. (**a**) Lanes M, molecular weight markers; lane 1, untagged hURAT1 wild type; lane 2, HA-tagged hURAT1 wild type, lane 3; HA-tagged hURAT1-S35N; lane 4, HA-tagged hURAT1-F365Y; lane 5, HA-tagged hURAT1-I481M; lane 6, HA-tagged hURAT1-R477K; lane 7, HA-tagged hURAT1-F449Y; lane 8, HA-tagged hURAT1-F241Y; lane 9, HA-tagged hURAT1 wild type; lane 10, untagged hURAT1 wild type. (**b**) Lane 1, HA-tagged hURAT1 wild type; lane 2, HA-tagged hURAT1-S35N; lane 3, HA-tagged hURAT1-F365Y; lane 4, HA-tagged hURAT1-S35N/F365Y; lane 5, HA-tagged rURAT1 wild type; lane 6, HA-tagged rURAT1-N35S; lane 7, HA-tagged rURAT1-Y365F; lane 8, HA-tagged rURAT1-N35S/Y365F; lanes M, molecular weight markers. All transporters were equivalently expressed. The difference in mobility between hURAT1 and rURAT1 is likely due to differences in glycosylation.

Supplementary Figure 1.

a

```
                            TMS 1                                        EC1
Human       MAFSELLDLVGGLGRFQVLQTMALMVSIMWLCTQSMLENFSAAVPSHRCWAPLLDNSTAQASILGSLSPEALLAISIPPGPNQRPHQCRRFRQPQWQLLDPNATATSWSEADTEPCVDGW  120
Consensus   MAF ELLD VGG GRFQ+LQ +AL+  I+W+ TQ+MLENFSAAVP HRCW PLLDNST+QASI G     + LLA+SIPPGP+QRPHQC RFRQPQWQL++ N TAT+WS+ADTEPC DGW
Rat         MAFPELLDRVGGRGRFQLLQAVALVTPILWVTTQNMLENFSAAVPHHRCWVPLLDNSTSQASIPGDFGRDVLLAVSIPPGPDQRPHQCLRFRQPQWQLIESNTTATNWSDADTEPCEDGW  120

                                      TMS 2                  TMS 3                     TMS 4              TMS 5
Human       VYDRSIFTSTIVAKWNLVCDSHALKPMAQSIYLAGILVGAAACGPASDRFGRRLVLTWSYLQMAVMGTAAAFAPAFPVYCLFRFLLAFAVAGVMMNTGTLLMEWTAARARPLVMTLNSLG  240
Consensus   VYD S F STIV  W+LVCDS AL+PMAQSI+LAGILVGAA CG ASDRFGRR VLTWSYL ++V GT AA  P FP+YCLFRFL+A AVAGVMMNT +LLMEWT+A+A PL+MTLN+LG
Rat         VYDHSTFRSTIVTTWDLVCDSQALRPMAQSIFLAGILVGAAVCGHASDRFGRRRVLTWSYLLVSVSGTIAALMPTFPLYCLFRFLVASAVAGVMMNTASLLMEWTSAQAGPLMMTLNALG  240

  TMS 5                 TMS 6                            IC3                                      TMS 7
Human       FSFGHGLTAAVAYGVRDWTLLQLVVSVPFFLCFLYSWWLAESARWLLTTGRLDWGLQELWRVAAINGKGAVQDTLTPEVLLSAMREELSMGQPPASLGTLLRMPGLRFRTCISTLCWFAF  360
Consensus   FSFG  LT +VAYGVR W +LQL VS PFFL F+YSWWL ESARWL+T GRLD  L+EL RVAA+N + A  DTLT EVL SAM+EE +  Q  A LGTLL  PGLR RT IS LCWFAF
Rat         FSFGQVLTGSVAYGVRSWRMLQLAVSAPFFLFFVYSWWLPESARWLITVGRLDQSLRELQRVAAVNRRKAEADTLTVEVLRSAMQEEPNGNQAGARLGTLLHTPGLRLRTFISMLCWFAF  360

  TMS 7                 TMS 8                    TMS 9                    TMS 10                 TMS 11
Human       GFTFFGLALDLQALGSNIFLLQMFIGVVDIPAKMGALLLLSHLGRRPTLAASLLLAGLCILANTLVPHEMGALRSALAVLGLGGVGAAFTCITIYSSELFPTVLRMTAVGLGQMAARGGA  480
Consensus   GFTF+GLALDLQALGSNIFLLQ  IG+VD+P KMG+LLLLS LGRR  A+SL+L GLCILAN LVP EMG LRS+LAVLGLG +GAAFTC+TI+SSELFPTV+RMTAVGLGQ+AARGGA
Rat         GFTFYGLALDLQALGSNIFLLQALIGIVDLPVKMGSLLLLSRLGRRLCQASSLVLPGLCILANILVPREMGILRSSLAVLGLGSLGAAFTCVTIFSSELFPTVIRMTAVGLGQVAARGGA  480

TMS 11          TMS 12
Human       ILGPLVRLLGVHGPWLPLLVYGTVPVLSGLAALLLPETQSLPLPDTIQDVQNQAVKKATHGTLGNSVLKSTQF  553
Consensus   +LGPLVRLLGV+G WLPLLVYG VPVLSGLAALLLPET++LPLPDTIQD+Q Q+VKK TH   G SVLKS +
Rat         MLGPLVRLLGVYGSWLPLLVYGVVPVLSGLAALLLPETKNLPLPDTIQDIQKQSVKKVTHDIAGGSVLKSARL  553
```

b

```
                                     35
                           TMS 1      ↓                                   EC1
URAT1 1     MAFSELLDLVGGLGRFQVLQTMALMVSIMWLCTQSMLENFSAAVPSHRCWAPLLDNSTAQASILGSLSPEALLAISIPPGPNQRPHQCRRFRQPQWQLLDPNATATSWSEADTEPCVDGW  120
Consensus   MAF++LL  VGG+GRFQ +Q   +++ ++ + + + L+NF+AA+P+H C  P   N          LS   L + +P     +P  C RF  PQW L   N T  + + A TEPC DGW
OAT1 1      MAFNDLLQQVGGVGRFQQIQVTLVVLPLLLMASHNTLQNFTAAIPTHHCRPPADAN----------LSKNGGLEVWLPRDRQGQPESCLRFTSPQWGLPFLNGTEANGTGA-TEPCTDGW  109

                                      TMS 2                  TMS 3                     TMS 4              TMS 5
URAT1       VYDRSIFTSTIVAKWNLVCDSHALKPMAQSIYLAGILVGAAACGPASDRFGRRLVLTWSYLQMAVMGTAAAFAPAFPVYCLFRFLLAFAVAGVMMNTGTLLMEWTAARARPLVMTLNSLG  240
Consensus   +YD S F STIV +W+LVC    AL+ +AQS+Y+ G+L+GA   G  +DR GRR VL  +YLQ AV GT AAFAP FP+YC FR L   A+AG+ +N  TL +EW      R  V TL
OAT1        IYDNSTFPSTIVTEWDLVCSHRALRQLAQSLYMVGVLLGAMVFGYLADRLGRRKVLILNYLQTAVSGTCAAFAPNFPIYCAFRLLSGMALAGISLNCMTLNVEWMPIHTRACVGTLIGYV  229

  TMS 5                 TMS 6                            IC3                                      TMS 7
URAT1       FSFGHGLTAAVAYGVRDWTLLQLVVSVPFFLCFLYSWWLAESARWLLTTGRLDWGLQELWRVAAINGKGAVQDTLTPEVLLSAMREELSMGQPPASLGTLLRMPGLRFRTCISTLCWFAF  360
Consensus   +S G  L A VAY V  W  LQL+VS PFF  F+YSW+ ESARW  ++GRLD  L+ L RVA INGK       L+ EVL +++++EL+MG+  AS    LLR P LR       ++ WFA
OAT1        YSLGQFLLAGVAYAVPHWRHLQLLVSAPFFAFFIYSWFFIESARWHSSSGRLDLTLRALQRVARINGKREEGAKLSMEVLRASLQKELTMGKGQASAMELLRCPTLRHLFLCLSMLWFAT  349
   365
    ↓                    TMS 8                    TMS 9                    TMS 10                 TMS 11
URAT1       GFTFFGLALDLQALGSNIFLLQMFIGVVDIPAKMGALLLLSHLGRRPTLAASLLLAGLCILANTLVPHEMGALRSALAVLGLGGVGAAFTCITIYSSELFPTVLRMTAVGLGQMAARGGA  480
Consensus    F ++GL +DLQ  G +I+L+Q+  G VD+PAK+   L+++ LGRRP  A+LLLAG+CIL N ++P +   +R++LAVLG G + A+F CI +Y+ EL+PT++R T +G+G   AR G+
OAT1        SFAYYGLVMDLQGFGVSIYLIQVIFGAVDLPAKLVGFLVINSLGRRPAQMAALLLAGICILLNGVIPQDQSIVRTSLAVLGKGCLAASFNCIFLYTGELYPTMIRQTGMGMGSTMARVGS  469
481
 ↓              TMS 12
URAT1       ILGPLVRLLGVHGPWLPLLVYGTVPVLSGLAALLLPETQSLPLPDTIQDVQNQAVKKATHGTLGNSVLKSTQF  553
Consensus   I+ PLV +      P +PL +YG VPV +     +LLPET  PLPDT+QD++++ K+             +   Q
OAT1        IVSPLVSMTAELYPSMPLFIYGAVPVAASAVTVLLPETLGQPLPDTVQDLESRKGKQTRQQQEHQKYMVPLQASAQEKNGL  550
```

Supplementary Figure 2.

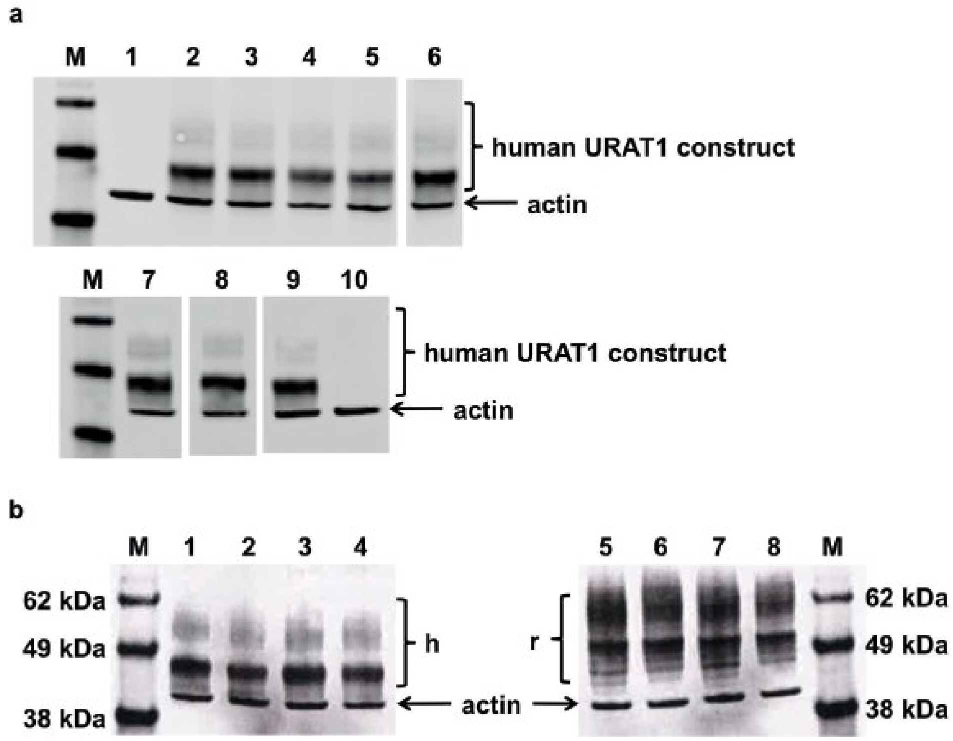

SUPPLEMENTARY TABLES

Supplementary Table 1. Mutagenic primers used in this study.
Residues in bold are modified from wild type sequences. Nucleotide positions correspond to those for coding sequences of the genes, so that the first ATG (initiator methionine) codon corresponds to nucleotides 1-3.

<table>
<tr><th>Name</th><th>Sequence</th><th>Purpose</th><th></th></tr>
<tr><td colspan="3">Primers for Production of h-r TM1 and r-h TM1 Chimeras</td><td></td></tr>
<tr><td>5’-ext F</td><td>GCAGAGCTCGTTTAGTGAACCGTCAG</td><td>Paired with int R for PCR</td><td></td></tr>
<tr><td>int R</td><td>CTGCCACTGAGGTTGGCGGAAGCG</td><td>Paired with 5’-ext F for PCR</td><td></td></tr>
<tr><td>int F</td><td>CGCTTCCGCCAACCTCAGTGGCAG</td><td>Paired with 3’-ext R for PCR</td><td></td></tr>
<tr><td>3’-ext R</td><td>GGGATGCCACCCGGGATCTG</td><td>Paired with int F for PCR</td><td></td></tr>
<tr><td colspan="3">Primers for Silent Mutagenesis and the Creation of Restriction Enzyme Sites</td><td>Notes</td></tr>
<tr><td>h-ClaI</td><td>GCTGTGTACCCAATCGATGCTGGAGAAC</td><td rowspan="2">Creates a ClaI site at nucleotides 102-107 (coding for amino acids 34-35 near the end of TM1)</td><td rowspan="4">h-r EC1,TMS 2-4 was produced from subclonings of URAT1 constructs containing engineered AscI and ClaI sites</td></tr>
<tr><td>r-ClaI</td><td>TTTGTGGGTCACCACCCAATCGATGCTGG AGAACTTCTCAGC</td></tr>
<tr><td>h-AscI</td><td>GGCGGCACGGGCGCGCCCCTTGGTGATG</td><td rowspan="2">Creates a AscI site at nucleotides 684-692 (coding for amino acids 229-231 between TMS 4 and TMS 5)</td></tr>
<tr><td>r-AscI</td><td>GTGGACATCAGCCCAGGCGCGCCCCTTGA TGATGACCTTGAACG</td></tr>
<tr><td>h-NheI-1</td><td>CTCCTGGTGGCTAGCAGAGTCGGCAC</td><td rowspan="2">Creates a NheI site at nucleotides 834-839, (coding for amino acids 278-280 at the end of TM6)</td><td rowspan="3">h-r IC3 was produced from subclonings of URAT1 constructs containing engineered NheI (from the NheI-1 primers) and BspEI sites (hURAT1 has a native BspEI site)</td></tr>
<tr><td>r-NheI-1</td><td>GTTTACTCATGGTGGCTAGCAGAATCAGC ACGCTGG</td></tr>
<tr><td>r-BspEI</td><td>CCTGGGCTGCGCCTCCGGACCTTCATCTC CATGCTG</td><td>Creates a BspEI site at nucleotides 1043-1048 (coding for amino acids 348-350 at the beginning of TMS 7)</td></tr>
</table>

h-HindIII	CCTGGACCTGCA**A**GC**TT**TGGGCAGCAACATC	Creates a *Hind*III site at nucleotides 1205-1210 (coding for amino acids 372-373 between TMS 7 and TMS 8)	h-r TMS 7 and r-h TMS 7 were produced from subclonings of URAT1 constructs containing engineered *Bsp*EI and *Hind*III sites (hURAT1 has a native BspEI site)
r-HindIII	GCCCTTGACCTGCAAGCT**TT**AGGAAGCAATATCTTCC		
h-NheI-2	CCGCCCCACGCT**A**GCCGCATCCCTG	Creates NheI site at nucleotides 1224-1229 (coding for amino acids 408-410 at beginning of TM9)	h-r TMS 8 was produced from subclonings of URAT1 constructs containing engineered *Hind*III and *Nhe*I (from the NheI-2 primers) sites
r-NheI-2	CCTCTGCCAGGC**T**AGCTCCCTGGTG		
**Primers for Production of Other Chimeras**			
h-TMS 5-N	GCACGGGCCCGACCCTTG**A**TGATGACCTTGAA**TG**CTCTGGGCTTCAGCTTCGGC	Production of h-r TMS 5-N, marked with *Bsm*I site	
h-TMS 5-C	CTGGGCTTCAGCTTCGGCCA**A**GTCCTGAC**C**G**G**T**T**CAGTGGCCTACGGTGTGCGG	Production of h-r TMS 5-C, marked with *Age*I site	
h-TM6	GTCCCCTTCTTCCTCT**T**CTTT**G**TATACTCCTGGTGGCTGGCAG	Production of h-r TM6, marked with AccI site	
h-TM9-N	CGCCGCCCCACGCTGGCC**T**CATCCCTG**G**TGCT**Cc**CGGGGCTCTGCATTCTGGC	Production of h-r TM9-N, marked with XmaI site	
h-TM9-C	TCTGCATTCTGGCCAACA**tc**CTGGTGCCCC**G**CGAAATGGGG**atC**CTGCGCTCA**T**CCTTGGCCGTGCTGGGGC	Production of h-r TM9-C, marked with BamHI site	
h-TM10	GCCGTGCTGGGGCTGGGC**tccc**TGGGGGCTGCCTTCAC**G**TGC**G**TCACCATCT**T**CAGCAGCGAGCTCTTCCC	Production of h-r TM10, marked with PmlI site	
h-TM11	GCAGTGGGCTTGGGCCAG**G**TGGCAGCCCG**G**GGAGGAGCCAT**G**CTGGGGCCTCTGGTCCGG	Production of h-r TM11, marked with XmaI site	
h-TM12	GTCCGGCTGCTGGGTGTC**T**ATGG**AT**CCTGGCTGCCCTTGCTGGTGTATGGG**gt**GGTGCCAGTGCTGAGTGG	Production of h-r TM12, marked with BamHI site	

h-V18L	CTGGGCAGGTTCCAG**C**T**G**CTCCAGACGAT GGCTC	Production of hURAT1-V18L, marked with *Pvu*II site
h-T21A	GTTCCAGGTTCTCCAG**G**C**C**ATGGCTCTGA TGGTC	Production of hURAT1-T21A, marked with *Xcm*I site
h-M22V	CAGGTTCTCCAGACG**G**T**A**GC**G**CTGATGGT CTCCATC	Production of hURAT1-M22V, marked with *Afe*I site
h-M25V	CCAGGTTCTCCAGAC**C**ATGGCTCTG**G**TGG TCTCCATCATGTG	Production of hURAT1-M25V, marked with *Xcm*I/*Nco*I sites
h-V26T	CCAGACGATGGCTCT**C**ATG**AC**CTCCATCA TGTGGCTG	Production of hURAT1-V26T, marked with *BspH*I site
h-S27P	GATGGCTCTGATGGT**AC**CCATCATGTGGC TGTG	Production of hURAT1-S27P, marked with *Kpn*I site
h-M29L	GATGGCTCTGATGGTC**AGT**AT**AC**TGTGGC TGTGTACC	Production of hURAT1-M29L, marked with *Acc*I site
h-L31V	GTCTCCATCATGTGG**G**TGTG**C**ACCCAGAG CATGCTG	Production of hURAT1-L31V, marked with *ApaL*I site
h-C32T	GTCTCCATCATGTGG**T**TG**AC**TACCCAGAG CATGCTG	Production of hURAT1-C32T, marked with *Hinc*II site
h-S35N	ATGTGGCTGTGTACCCAGA**A**CATG**T**TGGA GAACTTCTCGGCC	Production of hURAT1-S35N; marked with *Afl*III site
h-C351F/T354M	GGACTGCGCTTCCGGACCT**T**TAT**A**TC**G**A**T** GTTGTGCTGGTTCGCCTTTGG	Production of hURAT1-C351F/T354M; marked with *Cla*I site
h-F365Y	CCTTTGGCTTCACCTTCT**A**CGG**G**CT**A**GCC CTGGACCTGCAGGCC	Production of hURAT1-F365Y; marked with *Nhe*I site
h-M474V	GCAGTGGGCTTGGGCCAG**GT**GGCAGCCC G**G**GGAGGAGCCATC	Production of hURAT1-M474V; marked with *Xma*I site
h-I481M	GCCCGTGGAGG**C**GCCAT**G**CTGGGGCCTC TGGTCCGG	Production of hURAT1-I481M; marked with *Kas*I site

r-TM1-N	CCGGGGCAGGTTCCAG**G**TCCTCCAG**ACC** **A**TGGCTCTG**A**TG**GT**CCCCATTTTGTGGGT CACC	Production of r-h TM1-N; marked with XcmI site
r-TM1-C	GCAGTGGCTCTGGTGAC**GT**CCATT**A**TGTG G**C**TC**TG**CACCCAGA**G**CATGCTGGAGAACT TCTC	Production of r-h TM1-C; marked with AatII site
r-P27S	GCAGTGGCTCTGGTGAC**GT**CCATTTTGTG GGTCACC	Production of rURAT1-P27S; marked with *Aat*II site
r-L29M	GTGGCTCTGGTGACCCC**A**ATT**A**TGTGGGT CACCACCCAG	Production of rURAT1-L29M; marked with *BstX*I site
r-V31L	GTGACCCCCATTTTGTGG**T**T**A**ACCACCCA GAACATGCTG	Production of rURAT1-V31L; marked with *Hinc*II site
r-T32C	GACCCCCATTTTGTGGGT**gtg**CACCCAGAA CATGCTGG	Production of rURAT1-T32C; marked with *ApaL*I site
r-N35S	TTTGTGGGTCACCACCCA**atcg**ATGCTGGA GAACTTCTC	Production of rURAT1-N35S; marked with *Cla*I site
r-F351C	GCTGCGCCTCCGAACCT**G**CAT**A**TC**G**ATGC TGTGCTGGTTTGC	Production of rURAT1-F351C; marked with *Cla*I site
r-M354T	CTCCGAACCTTCATCTC**A**A**C**G**T**TGTGCTG GTTTGCCTTTG	Production of rURAT1-M354T; marked with *Acl*I site
r-Y365F	CCTTTGGCTTCACCTTCT**T**TGG**G**CT**A**GCCC TTGACCTGCAAGC	Production of rURAT1-Y365F; marked with *Nhe*I site
r-TM11	cagtgggcctgggccag**at**g**g**gcagc**t**cgaggaggagccat **c**ctagggcctttggtgcgg	Production of r-h TM11; marked with XhoI site
r-V474M	GCAGTGGGCCTGGGCCAG**A**T**G**GCAGCCC G**G**GGAGGAGCCATGCTAGG	Production of rURAT1-V474M; marked with *Xma*I site
r-M481I	GCCCGAGGAGGAGCCAT**C**CTAGGGCC**AC** T**A**GTGCGGCTGCTGGGTGTC	Production of rURAT1-M481I; marked with *Spe*I site

Supplementary Table 2. Potencies of URAT1 inhibitors against human (h) URAT1, rat (r) URAT1, and chimeras. Numbers are the mean ± sem IC_{50} values in μM from at least three dose-response experiments, as shown in Figure 1. Values indicated in red are statistically significant from the corresponding wild type (WT) URAT1. Asterisks for rat WT show the statistically significant difference from human WT. * *P*< 0.05; ** *P* < 0.01; *** *P* < 0.001; **** *P* < 0.0001.

		URAT1 Inhibitor			
	construct	benzbromarone	sulfinpyrazone	probenecid	lesinurad
Human WT	hURAT1	0.22 ± 0.02	32 ± 5	22 ± 2.1	3.5 ± 0.32
rat WT	rURAT1	26 ± 1.2****	680 ± 54****	786 ± 57****	81 ± 4.0****

Human WT	hURAT1	0.22 ± 0.02	32 ± 5	22 ± 2.1	3.5 ± 0.32
Human to rat chimeras	h-r TM1	1.2 ± 0.20****	93 ± 17	14 ± 3.0*	4.8 ± 1.6
	h-r EC1,TM2-4	0.35 ± 0.13	3.4 ± 1.6	23 ± 5.6	1.1 ± 0.54**
	h-r TM5-N	0.20 ± 0.05	37 ± 9	27 ± 7.9	2.2 ± 0.80
	h-r TM5-C	0.14 ± 0.03*	34 ± 4	23 ± 0.76	8.5 ± 2.1***
	h-r TM6	0.27 ± 0.05	30 ± 4	34 ± 5.7*	6.8± 1.0***
	h-r IC3	0.35 ± 0.06*	67 ± 15	39 ± 4.3**	2.6 ± 0.66
	h-r TM7	5.1 ± 0.45****	254 ± 47	134 ± 17****	79 ± 8.3****
	h-r TM8	0.22 ± 0.05	29 ± 13	14 ± 1.4	2.5 ± 0.77
	h-r TM9-N	0.44 ± 0.09**	36 ± 17	87 ± 22**	9.2 ± 3.3***
	h-r TM9-C	0.27 ± 0.06	38 ± 11	26 ± 4.5	6.5 ± 1.4**
	h-r TM10	0.45 ± 0.05***	49 ± 11	82 ± 13***	4.4 ± 1.3
	h-r TM11	1.5 ± 0.21****	100 ± 15	160 ± 32***	16 ± 1.3****
	h-r TM12	0.30 ± 0.04	36 ± 8	39 ± 5.8**	3.9 ± 1.1

Rat WT	rURAT1	26 ± 1.2	680 ± 54	786 ± 57	81 ± 4.0
Rat to human chimeras	r-h TM1	12 ± 1.1***	87 ± 25***	353 ± 23**	18 ± 1.6****
	r-h TM1-N	16 ± 0.76***	12 ± 2.6****	337 ± 20**	40 ± 7.0***

	r-h TM1-C	12 ± 0.78***	25 ± 6.7****	151 ± 6.7***	17 ± 2.0****
	r-h TM7	5.7 ± 0.25****	243 ± 22***	298 ± 36***	6.9 ± 0.99****
	r-h TM11	12 ± 0.48***	342 ± 32**	272 ± 41***	62 ± 3.6**
Human WT	hURAT1	0.22 ± 0.02	32 ± 5	22 ± 2.1	3.5 ± 0.32
Human to rat point mutants in TM1	h-V18L	0.17 ± 0.05	19 ± 4.7	19 ± 0.94	2.2 ± 0.22
	h-T21A	0.16 ± 0.02	12 ± 2.3*	15 ± 0.66	2.2 ± 1.1
	h-M22V	0.26 ± 0.07	28 ± 3.1	19 ± 5.8	3.5 ± 1.0
	h-M25V	0.24 ± 0.05	53 ± 6.0*	14 ± 3.0	3.7 ± 0.59
	h-V26T	0.26 ± 0.05	21 ± 4.7	16 ± 3.0	3.9 ± 0.51
	h-S27P	0.17 ± 0.04	9.3 ± 1.7**	9.9 ± 0.85**	1.9 ± 0.63*
	h-M29L	0.27 ± 0.05	8.5 ± 4.4*	15 ± 2.4	4.8 ± 1.3
	h-L31V	0.23 ± 0.05	25 ± 2.9	4.9 ± 1.3***	5.3 ± 0.67*
	h-C32T	0.22 ± 0.04	29 ± 3.2	27 ± 4.5	2.9 ± 0.46
	h-S35N	2.1 ± 0.17****	233 ± 54***	13 ± 2.4*	5.0 ± 0.47*
Human to rat point mutants in TM7	h-C351F/T354M	0.59 ± 0.08****	60 ± 5.9**	61 ± 12**	6.3 ± 0.71***
	h-F365F	3.7 ± 0.66****	163 ± 13****	73 ± 14***	57 ± 6.1****
Human to rat point mutants in TM11	h-M474V	0.34 ± 0.03**	30 ± 3.4	32 ± 7.0	2.7 ± 0.26
	h-I481M	1.1 ± 0.12****	118 ± 8.1****	118 ± 26***	5.8 ± 0.54***
Rat WT	rURAT1	26 ± 1.2	680 ± 54	786 ± 57	81 ± 4.0
Rat to human point mutants in TM1	r-L18V	24 ± 3.1	971 ± 89*	770 ± 180	78 ± 6.6
	r-A21T	22 ± 3.6	471 ± 81	505 ± 91*	71 ± 5.9
	r-V22M	20 ± 2.7*	762 ± 177	658 ± 126	74 ± 8.9
	r-V25M	11 ± 2.7***	14 ± 2.3****	171 ± 28***	59 ± 17
	r-T26V	26 ± 4.4	655 ± 191	844 ± 227	76 ± 2.3
	r-P27S	16 ± 1.7**	321 ± 77**	408 ± 51**	77 ± 3.2
	r-L29M	21 ± 3.3	495 ± 152	513 ± 106*	77 ± 6.6
	r-V31L	17 ± 3.4*	266 ± 70**	564 ± 103	85 ± 4.5
	r-T32C	24 ± 4.0	691 ± 98	747 ± 189	73 ± 5.9
	r-N35S	17 ± 2.1**	189 ± 32***	264 ± 43***	57 ± 6.3**

Rat to human point mutants in TM7	r-F351C	24 ± 3.3	924 ± 276	725 ± 47	82 ± 1.7
	r-M354T	18 ± 1.7**	304 ± 38**	362 ± 20**	56 ± 4.9**
	r-Y365F	9.4 ± 1.6***	255 ± 27**	525 ± 76*	15 ± 3.0****
Rat to human point mutants in TM11	r-V474M	17 ± 2.2**	732 ± 102	567 ± 91	84 ± 3.2
	r-M481I	15 ± 1.1***	395 ± 68*	225 ± 13***	71 ± 3.0
Rat to human combination point mutants at residues 35, 365 and 481	r-N35S/Y365F	2.2 ± 0.29****	142 ± 15***	207 ± 37***	1.6 ± 0.03****
	r-N35S/M481I	9.0 ± 1.5****	176 ± 44***	146 ± 25***	29 ± 1.2****
	r-Y365F/M481I	3.5 ± 0.39****	138 ± 25***	213± 38***	5.5 ± 1.1****
	r-N35S/Y365F/M481I	0.61 ± 0.04****	49 ± 2.2****	62 ± 6.4****	1.2 ± 0.14****

Supplementary Table 3. List of non-conserved residues in the URAT1 chimeras shown in Figure 2.
Non-conserved residues are listed based on their order within the 553-residue proteins.

	construct	nonconserved residues
Human to rat chimeras	h-r TM1	4, 9, 13, 18, 21, 22, 25, 26, 27, 29, 31, 32, 35, 46, 51, 59, 64, 66, 67, 68, 69, 70, 71, 75, 82, 89, 99
	h-r EC1,TM2-4	46, 51, 59, 64, 66, 67, 68, 69, 70, 71, 75, 82, 89, 99, 100, 101, 103, 107, 110, 117, 124, 126, 128, 133, 134, 136,142, 145, 152, 162, 165, 174, 182, 183, 184, 186, 189, 192, 193, 195, 198, 206, 208, 218, 219, 226
	h-r TM5-N	233, 238
	h-r TM5-C	245, 246, 249, 250
	h-r TM6	272, 274
	h-r IC3	287, 289, 294, 295, 297, 300, 305, 307, 308, 309, 311, 312, 317, 321, 325, 328, 329, 330, 331, 333, 334, 336, 342, 343, 348
	h-r TM7	351, 354, 365
	h-r TM8	383, 384, 387, 390, 392, 396, 402, 407
	h-r TM9-N	411, 414, 416
	h-r TM9-C	424, 428, 432, 436
	h-r TM10	444, 445, 452, 455
	h-r TM11	474, 481
	h-r TM12	492, 494, 503
Rat to human chimeras	r-h TM1	4, 9, 13, 18, 21, 22, 25, 26, 27, 29, 31, 32, 35, 46, 51, 59, 64, 66, 67, 68, 69, 70, 71, 75, 82, 89, 99
	r-h TM1-N	18, 21, 22, 25, 26
	r-h TM1-C	27, 29, 31, 32, 35
	r-h TM7	351, 354, 365
	r-h TM11	474, 481

Supplementary Table 4. Identity of residues corresponding to human URAT1 residues 35, 365, and 481 in human URAT1 orthologs and homologs within the *SLC22A* transporter subfamily.

	Transporter	% identity	amino acid at the position corresponding to the hURAT1 residue		
			35	365	481
	human		S	F	I
ORTHOLOGS	chimpanzee	99	S	F	I
	gorilla	99	S	F	I
	orangutan	98	N	F	I
	macaque	95	S	F	I
	baboon	95	N	F	I
	squirrel monkey	88	N	F	I
	dog	83	S	Y	I
	pig	83	S	Y	I
	elephant	78	N	Y	I
	hamster	75	N	Y	I
	mole rat	75	N	Y	M
	mouse	74	N	Y	M
	rat	74	N	Y	M
	horse	74	N	Y	I
	guinea pig	72	N	Y	I
HOMOLOGS	*SLC22A11*/OAT4	51	M	Y	M
	SLC22A10/OAT5	51	I	Y	A
	SLC22A24	47	I	Y	A
	SLC22A25	47	T	W	A
	SLC22A6/OAT1	46	N	Y	I
	SLC22A9/OAT7	45	F	F	A
	SLC22A8/OAT3	42	N	Y	M
	SLC22A20	38	N	Y	L
	SLC22A7/OAT2	37	F	Y	S
	SLC22A13/OAT10	35	F	Y	I
	SLC22A1/OCT1	32	C	Q	I

	SLC22A2/OCT2	31	Y	Q	I
	SLC22A5/OCTN2	31	G	F	I
	SLC22A4/OCTN1	30	G	F	I
